# 2-(7,8-Diphenyl-1*H*-imidazo[4,5-*f*]quinoxalin-2-yl)phenol methanol disolvate

**DOI:** 10.1107/S1600536808025269

**Published:** 2008-08-13

**Authors:** Hoong-Kun Fun, Reza Kia, Paul R. Raithby

**Affiliations:** a X-ray Crystallography Unit, School of Physics, Universiti Sains Malaysia, 11800 USM, Penang, Malaysia; b Chemistry Department, University of Bath, Claverton Down, Bath BA2 7AY, England

## Abstract

The title compound, C_27_H_18_N_4_O·2CH_4_O, is a unsymmetrically substituted quinoxaline. An intra­molecular O—H⋯N hydrogen bond involving the hydr­oxy and imino groups generates an *S*(6) ring motif. Inter­molecular C—H⋯O and N—H⋯O hydrogen bonds form an *R*_2_^1^(7) ring motif involving a methanol O atom and two H atoms of the imidazole and benzene rings, respectively. The latter links neighbouring mol­ecules into one-dimensional extended chains along the *a* axis. The two benzene rings are inclined towards each other, as indicated by the dihedral angle of 52.13 (10)°. The phenol ring is almost coplanar with the basic quinoxaline unit, making a dihedral angle of 2.43 (6)°. The short distances between the centroids of the five- and six-membered rings prove the existence of π–π inter­actions [centroid–centroid distances = 3.5234 (9)–3.7885 (10) Å]. The crystal structure is stabilized by intra­molecular O—H⋯N, inter­molecular O—H⋯O, N—H⋯O and C—H⋯O (× 2) hydrogen bonds and weak inter­molecular C—H⋯π and π–π inter­actions.

## Related literature

For hydrogen-bond motifs, see: Bernstein *et al.* (1995 ▶). For bond-length data, see: Allen *et al.* (1987 ▶). For information about imidazolyl quinoxaline, see, for example: Mamedov *et al.* (2004 ▶); Miranda *et al.* (2008 ▶); Bhosale *et al.* (2005 ▶); Kanoktanaporn *et al.* (1980 ▶); Ali *et al.* (2000 ▶); Veroni *et al.* (2008 ▶); Zarranz *et al.* (2004 ▶); Addess *et al.* (1993 ▶); Mollegaard *et al.* (2000 ▶).
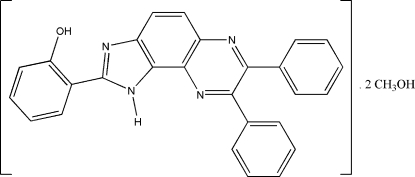


## Experimental

### 

#### Crystal data

C_27_H_18_N_4_O·2CH_4_O
*M* *_r_* = 478.54
Triclinic, 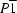

*a* = 10.5120 (3) Å
*b* = 11.4574 (2) Å
*c* = 11.9983 (2) Å
α = 116.325 (1)°
β = 107.465 (1)°
γ = 95.147 (1)°
*V* = 1192.81 (4) Å^3^
*Z* = 2
Mo *K*α radiation
μ = 0.09 mm^−1^
*T* = 100.0 (1) K
0.39 × 0.29 × 0.12 mm

#### Data collection

Bruker SMART APEXII CCD area-detector diffractometer
Absorption correction: multi-scan (**SADABS**; Bruker, 2005 ▶) *T*_min_ = 0.876, *T*_max_ = 0.990
23412 measured reflections
7076 independent reflections
5031 reflections with *I* > 2σ(*I*)
*R*_int_ = 0.043

#### Refinement

*R*[*F* ^2^ > 2σ(*F* ^2^)] = 0.061
*wR*(*F* ^2^) = 0.182
*S* = 1.07
7076 reflections
335 parameters
H atoms treated by a mixture of independent and constrained refinement
Δρ_max_ = 0.99 e Å^−3^
Δρ_min_ = −0.48 e Å^−3^

### 

Data collection: *APEX2* (Bruker, 2005 ▶); cell refinement: *APEX2*; data reduction: *SAINT* (Bruker, 2005 ▶); program(s) used to solve structure: *SHELXTL* (Sheldrick, 2008 ▶); program(s) used to refine structure: *SHELXTL*; molecular graphics: *SHELXTL*; software used to prepare material for publication: *SHELXTL* and *PLATON* (Spek, 2003 ▶).

## Supplementary Material

Crystal structure: contains datablocks global, I. DOI: 10.1107/S1600536808025269/at2610sup1.cif

Structure factors: contains datablocks I. DOI: 10.1107/S1600536808025269/at2610Isup2.hkl

Additional supplementary materials:  crystallographic information; 3D view; checkCIF report

## Figures and Tables

**Table 1:** Selected centroid⋯centroid distances (Å)

*Cg*1⋯*Cg*1^i^	3.7885 (10)
*Cg*2⋯*Cg*3^i^	3.5234 (7)
*Cg*3⋯*Cg*4^i^	3.6348 (11)

Symmetry code: (i) 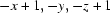. *Cg*1, *Cg*2, *Cg*3 and *Cg*4 are the centroids of the N1/N4/C7/C8/C15, N2/N3/C11–C14, C1–C6 and C8–C15 benzene rings, respectively.

**Table 2:** Hydrogen-bond geometry (Å, °)

*D*—H⋯*A*	*D*—H	H⋯*A*	*D*⋯*A*	*D*—H⋯*A*
O2—H1*O*2⋯O3	1.00	1.71	2.700 (3)	167
O3—H1*O*3⋯N3^iii^	0.95	1.87	2.814 (2)	172
N4—H1*N*4⋯O2	0.97 (3)	1.78 (3)	2.750 (2)	177 (2)
O1—H1*O*1⋯N1	0.97 (4)	1.66 (4)	2.570 (2)	154 (3)
C2—H2*A*⋯O1^iv^	0.93	2.48	3.356 (3)	156
C5—H5*A*⋯O2	0.93	2.42	3.310 (3)	160
C28—H28*C*⋯*Cg*5^v^	0.96	2.95	3.534 (2)	120

Symmetry codes: (iii) 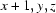; (iv) 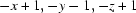; (v) 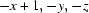. *Cg*5 is the centroid of the C22–C27 benzene ring.

**Fig. 1.**
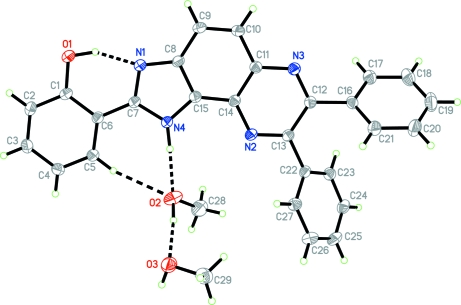
The molecular structure of the title compound, showing 50% probability displacement ellipsoids and the atomic numbering. Intramolecular and intermolecular interactions are drawn as dashed lines.

**Fig. 2.**
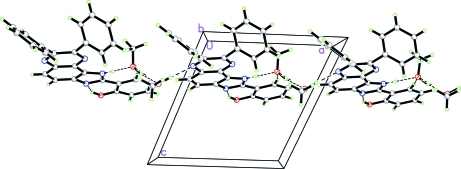
The crystal packing of (I), viewed down the b-axis, showing an 1-D extended chain along the a-axis. Intramolecular and intermolecular interactions are drawn as dashed lines.

## supplementary crystallographic information

### Comment

Quinoxaline structure is recognized in a growing number of naturally occurring compounds such as riboflavin (vitamin B2), flavoenzymes, molybdopterines and antibiotics of Streptomyces (Ali *et al.*, 2000; Veroni *et al.*, 2008). Quinoxaline derivatives have already been used as antibacterial, antiviral, anticancer, antifungal, antihelmintic and insecticidal agents (Zarranz *et al.*, 2004). The widely prescribed quinoxaline antibiotics are found to bind specifically by bisintercalation to double-stranded DNA (Addess *et al.*, 1993) and to enhance peptide nucleic acid (PNA) binding to it (Mollegaard *et al.*, 2000), stimulating the research on the DNA-interactive ligands. In addition, some disubstituted quinoxaline derivatives have been found as potent antagonists of the quisqualate and kainate receptors on neurones of the central nervous system. To the best of our knowledge, this compound is the first quinoxaline with both phenol and imidazole substituents. In view of the importance of these organic ligands, the title compound (I) was synthesized and its crystal structure is repoted here.

The bond lenghts and angles are in normal ranges (Allen *et al.*, 1987). An intramolecular O—H···N hydrogen bond involving the hydroxy and the N atom of the imidazole group generate *S*(6) ring motif (Bernstein *et al.* 1995). An intermolecular C—H···O and N—H···O hydrogen bonds form an *R*_2_^1^(7) ring motif involving an oxygen of the methanol and two H atoms of the imidazole and benzene rings, respectively (Bernstein *et al.* 1995). The latter links neighbouring molecules into 1-D extended chains (Fig. 2) along the *a* axis. The two benzene rings are inclined to each other and their orientations are shown by the dihedral angle of 52.13 (10) °. The phenol ring is almost coplanar with the basic quinoxaline unit making the dihedral angle of 2.43 (6) °. The short distances between the centroids of the five and six-membered rings prove an existence of π-π interactions (Table 1) [centroid–centroid distances ranging from 3.5234 (9) to 3.7885 (10) Å]. The crystal structure is stabilized by intramolecular O—H···N, intermolecular O—H···O, N—H···O, C—H···O (*x* 2) hydrogen bonds, weak intermolecular C—H···π and π-π interactions.

### Experimental

A mixture of (*E*)-2-((5-amino-2,3-diphenylquinoxalin-6-ylimino)methyl) -phenol (418 mg, 1 mmol) in 20 ml of dichloromethane was added to a 20 ml methanolic solution of CoCl_2_. 6H_2_O (238 mg, 1 mmol). The reaction mixture was stirred under heating/boiling condition for 1 h. After cooling, the brown crystalline products was filtered, washed with ethanol and ether and then dried at room temperature.

### Refinement

The H-atoms attached to O1 and N4 were located from the difference Fourier map and refined freely. The H-atoms attached to O2 and O3 were located from the difference Fourier map and then costrained to ride on the parent atoms with an isotropic displacement parameter 1.5 times that of the parent atom. The rest of the hydrogen atoms were positioned geometrically [C—H = 0.93 - 0.96 Å] and refined using a riding model. A rotating-group model was applied for the methyl groups.

### Crystal data

**Table:**

C_27_H_18_N_4_O_1_·2C_1_H_4_O_1_	*V* = 1192.81 (4) Å^3^
*M**_r_* = 478.54	*Z* = 2
Triclinic, *P*1	*F*_000_ = 504
Hall symbol: -P 1	*D*_x_ = 1.332 Mg m^−^^3^
*a* = 10.5120 (3) Å	Mo *K*α radiation λ = 0.71073 Å
*b* = 11.4574 (2) Å	Cell parameters from 4368 reflections
*c* = 11.9983 (2) Å	µ = 0.09 mm^−^^1^
α = 116.325 (1)º	*T* = 100.0 (1) K
β = 107.465 (1)º	Block, brown
γ = 95.147 (1)º	0.39 × 0.29 × 0.12 mm

### Data collection

**Table:**

Bruker SMART APEXII CCD area-detector diffractometer	7076 independent reflections
Radiation source: fine-focus sealed tube	5031 reflections with *I* > 2σ(*I*)
Monochromator: graphite	*R*_int_ = 0.043
*T* = 100.0(1) K	θ_max_ = 30.3º
φ and ω scans	θ_min_ = 2.1º
Absorption correction: multi-scan(*SADABS*; Bruker, 2005)	*h* = −14→14
*T*_min_ = 0.876, *T*_max_ = 0.990	*k* = −16→16
23412 measured reflections	*l* = −16→16

### Refinement

**Table:**

Refinement on *F*^2^	Secondary atom site location: difference Fourier map
Least-squares matrix: full	Hydrogen site location: inferred from neighbouring sites
*R*[*F*^2^ > 2σ(*F*^2^)] = 0.061	H atoms treated by a mixture of independent and constrained refinement
*wR*(*F*^2^) = 0.182	*w* = 1/[σ^2^(*F*_o_^2^) + (0.0919*P*)^2^ + 0.4066*P*] where *P* = (*F*_o_^2^ + 2*F*_c_^2^)/3
*S* = 1.07	(Δ/σ)_max_ < 0.001
7076 reflections	Δρ_max_ = 0.99 e Å^−^^3^
335 parameters	Δρ_min_ = −0.48 e Å^−^^3^
Primary atom site location: structure-invariant direct methods	Extinction correction: none

### Special details

**Table:**

Experimental. The low-temperature data was collected with the Oxford Cyrosystem Cobra low-temperature attachment.
Geometry. All e.s.d.'s (except the e.s.d. in the dihedral angle between two l.s. planes) are estimated using the full covariance matrix. The cell e.s.d.'s are taken into account individually in the estimation of e.s.d.'s in distances, angles and torsion angles; correlations between e.s.d.'s in cell parameters are only used when they are defined by crystal symmetry. An approximate (isotropic) treatment of cell e.s.d.'s is used for estimating e.s.d.'s involving l.s. planes.
Refinement. Refinement of *F*^2^ against ALL reflections. The weighted *R*-factor *wR* and goodness of fit *S* are based on *F*^2^, conventional *R*-factors *R* are based on *F*, with *F* set to zero for negative *F*^2^. The threshold expression of *F*^2^ > σ(*F*^2^) is used only for calculating *R*-factors(gt) *etc*. and is not relevant to the choice of reflections for refinement. *R*-factors based on *F*^2^ are statistically about twice as large as those based on *F*, and *R*- factors based on ALL data will be even larger.

### Fractional atomic coordinates and isotropic or equivalent isotropic displacement parameters (Å^2^)

**Table:**

	*x*	*y*	*z*	*U*_iso_*/*U*_eq_	
O1	0.43178 (14)	−0.35511 (14)	0.48044 (14)	0.0269 (3)	
N1	0.31793 (14)	−0.20440 (14)	0.39691 (14)	0.0206 (3)	
N2	0.29306 (14)	0.09233 (14)	0.22570 (14)	0.0190 (3)	
N3	0.03863 (14)	0.11984 (14)	0.25061 (14)	0.0201 (3)	
N4	0.42131 (14)	−0.09206 (14)	0.32161 (14)	0.0194 (3)	
C1	0.53792 (17)	−0.32830 (17)	0.44611 (16)	0.0204 (3)	
C2	0.64738 (18)	−0.38570 (17)	0.47042 (17)	0.0230 (3)	
H2A	0.6462	−0.4398	0.5096	0.028*	
C3	0.75724 (18)	−0.36215 (18)	0.43628 (18)	0.0247 (4)	
H3A	0.8303	−0.4001	0.4533	0.030*	
C4	0.76027 (18)	−0.28222 (19)	0.37661 (18)	0.0243 (4)	
H4A	0.8341	−0.2682	0.3525	0.029*	
C5	0.65261 (17)	−0.22384 (18)	0.35341 (17)	0.0220 (3)	
H5A	0.6550	−0.1699	0.3143	0.026*	
C6	0.54036 (16)	−0.24495 (16)	0.38802 (16)	0.0191 (3)	
C7	0.42786 (17)	−0.18162 (16)	0.36860 (16)	0.0189 (3)	
C8	0.23639 (17)	−0.12638 (16)	0.36740 (16)	0.0197 (3)	
C9	0.10836 (17)	−0.11210 (17)	0.37990 (18)	0.0223 (3)	
H9A	0.0684	−0.1577	0.4137	0.027*	
C10	0.04485 (18)	−0.02999 (17)	0.34120 (18)	0.0222 (3)	
H10A	−0.0393	−0.0193	0.3490	0.027*	
C11	0.10607 (16)	0.03970 (16)	0.28893 (16)	0.0190 (3)	
C12	0.09500 (16)	0.18353 (16)	0.20112 (16)	0.0186 (3)	
C13	0.22417 (16)	0.16765 (16)	0.18646 (16)	0.0182 (3)	
C14	0.23573 (16)	0.02835 (16)	0.27780 (16)	0.0184 (3)	
C15	0.29957 (17)	−0.05638 (16)	0.31951 (16)	0.0192 (3)	
C16	0.01785 (17)	0.27117 (17)	0.16282 (17)	0.0204 (3)	
C17	−0.12304 (18)	0.21913 (19)	0.08189 (18)	0.0256 (4)	
H17A	−0.1672	0.1312	0.0538	0.031*	
C18	−0.1971 (2)	0.2984 (2)	0.0433 (2)	0.0334 (4)	
H18A	−0.2905	0.2630	−0.0116	0.040*	
C19	−0.1323 (2)	0.4301 (2)	0.0864 (2)	0.0383 (5)	
H19A	−0.1819	0.4828	0.0600	0.046*	
C20	0.0072 (2)	0.4835 (2)	0.1693 (2)	0.0343 (4)	
H20A	0.0502	0.5724	0.1996	0.041*	
C21	0.0823 (2)	0.40404 (18)	0.20673 (19)	0.0265 (4)	
H21A	0.1758	0.4396	0.2612	0.032*	
C22	0.28550 (17)	0.23115 (16)	0.12362 (16)	0.0192 (3)	
C23	0.20742 (18)	0.21654 (18)	−0.00052 (17)	0.0228 (3)	
H23A	0.1148	0.1690	−0.0437	0.027*	
C24	0.2674 (2)	0.27272 (19)	−0.05977 (18)	0.0259 (4)	
H24A	0.2151	0.2613	−0.1434	0.031*	
C25	0.4040 (2)	0.34550 (19)	0.00426 (19)	0.0281 (4)	
H25A	0.4436	0.3834	−0.0358	0.034*	
C26	0.4826 (2)	0.36194 (19)	0.12905 (19)	0.0278 (4)	
H26A	0.5745	0.4117	0.1729	0.033*	
C27	0.42355 (18)	0.30390 (18)	0.18805 (17)	0.0229 (3)	
H27A	0.4765	0.3137	0.2707	0.028*	
O2	0.62482 (16)	0.01159 (16)	0.26543 (16)	0.0394 (4)	
H1O2	0.7056	0.0933	0.3188	0.059*	
C28	0.5765 (2)	0.0070 (2)	0.1395 (2)	0.0398 (5)	
H28A	0.5578	0.0915	0.1519	0.060*	
H28B	0.4933	−0.0643	0.0802	0.060*	
H28C	0.6455	−0.0093	0.1012	0.060*	
O3	0.86046 (15)	0.20926 (15)	0.38666 (16)	0.0366 (3)	
H1O3	0.9203	0.1858	0.3389	0.055*	
C29	0.8398 (2)	0.3352 (2)	0.4050 (2)	0.0352 (4)	
H29A	0.8729	0.3989	0.4994	0.053*	
H29B	0.7430	0.3260	0.3642	0.053*	
H29C	0.8893	0.3665	0.3638	0.053*	
H1N4	0.492 (3)	−0.053 (2)	0.302 (2)	0.038 (6)*	
H1O1	0.370 (3)	−0.303 (3)	0.460 (3)	0.054 (8)*	

### Atomic displacement parameters (Å^2^)

**Table:**

	*U*^11^	*U*^22^	*U*^33^	*U*^12^	*U*^13^	*U*^23^
O1	0.0281 (6)	0.0313 (7)	0.0364 (7)	0.0112 (5)	0.0177 (6)	0.0248 (6)
N1	0.0209 (7)	0.0221 (7)	0.0245 (7)	0.0066 (5)	0.0106 (6)	0.0146 (6)
N2	0.0215 (7)	0.0202 (7)	0.0182 (6)	0.0051 (5)	0.0090 (5)	0.0111 (5)
N3	0.0213 (7)	0.0201 (7)	0.0202 (7)	0.0055 (5)	0.0087 (5)	0.0105 (6)
N4	0.0202 (6)	0.0226 (7)	0.0216 (7)	0.0074 (5)	0.0098 (5)	0.0145 (6)
C1	0.0228 (8)	0.0198 (8)	0.0193 (7)	0.0028 (6)	0.0083 (6)	0.0105 (6)
C2	0.0263 (8)	0.0219 (8)	0.0228 (8)	0.0069 (6)	0.0071 (7)	0.0140 (7)
C3	0.0231 (8)	0.0260 (9)	0.0254 (8)	0.0082 (7)	0.0069 (7)	0.0141 (7)
C4	0.0201 (8)	0.0305 (9)	0.0256 (8)	0.0065 (7)	0.0096 (7)	0.0161 (7)
C5	0.0215 (8)	0.0260 (8)	0.0223 (8)	0.0059 (6)	0.0089 (6)	0.0147 (7)
C6	0.0198 (7)	0.0200 (7)	0.0187 (7)	0.0044 (6)	0.0069 (6)	0.0109 (6)
C7	0.0207 (7)	0.0193 (7)	0.0180 (7)	0.0040 (6)	0.0077 (6)	0.0104 (6)
C8	0.0205 (7)	0.0198 (8)	0.0212 (8)	0.0039 (6)	0.0087 (6)	0.0118 (6)
C9	0.0226 (8)	0.0239 (8)	0.0263 (8)	0.0053 (6)	0.0130 (7)	0.0151 (7)
C10	0.0210 (8)	0.0247 (8)	0.0260 (8)	0.0067 (6)	0.0131 (7)	0.0139 (7)
C11	0.0198 (7)	0.0194 (7)	0.0183 (7)	0.0052 (6)	0.0079 (6)	0.0092 (6)
C12	0.0199 (7)	0.0190 (7)	0.0173 (7)	0.0057 (6)	0.0073 (6)	0.0090 (6)
C13	0.0197 (7)	0.0182 (7)	0.0166 (7)	0.0040 (6)	0.0067 (6)	0.0087 (6)
C14	0.0208 (7)	0.0192 (7)	0.0170 (7)	0.0053 (6)	0.0083 (6)	0.0097 (6)
C15	0.0201 (7)	0.0208 (8)	0.0192 (7)	0.0058 (6)	0.0088 (6)	0.0108 (6)
C16	0.0227 (8)	0.0235 (8)	0.0204 (8)	0.0107 (6)	0.0115 (6)	0.0122 (7)
C17	0.0251 (8)	0.0276 (9)	0.0245 (8)	0.0086 (7)	0.0089 (7)	0.0132 (7)
C18	0.0288 (9)	0.0418 (11)	0.0305 (10)	0.0166 (8)	0.0081 (8)	0.0192 (9)
C19	0.0429 (12)	0.0403 (12)	0.0442 (12)	0.0252 (10)	0.0182 (10)	0.0275 (10)
C20	0.0423 (11)	0.0266 (9)	0.0430 (11)	0.0150 (8)	0.0196 (9)	0.0213 (9)
C21	0.0288 (9)	0.0237 (8)	0.0298 (9)	0.0095 (7)	0.0129 (7)	0.0140 (7)
C22	0.0233 (8)	0.0187 (7)	0.0203 (8)	0.0076 (6)	0.0110 (6)	0.0113 (6)
C23	0.0238 (8)	0.0249 (8)	0.0225 (8)	0.0078 (7)	0.0094 (7)	0.0133 (7)
C24	0.0334 (9)	0.0288 (9)	0.0223 (8)	0.0096 (7)	0.0117 (7)	0.0170 (7)
C25	0.0378 (10)	0.0259 (9)	0.0283 (9)	0.0061 (7)	0.0169 (8)	0.0172 (8)
C26	0.0283 (9)	0.0262 (9)	0.0265 (9)	0.0001 (7)	0.0114 (7)	0.0116 (7)
C27	0.0248 (8)	0.0252 (8)	0.0201 (8)	0.0053 (7)	0.0091 (6)	0.0121 (7)
O2	0.0436 (8)	0.0401 (8)	0.0442 (9)	0.0048 (7)	0.0230 (7)	0.0259 (7)
C28	0.0470 (12)	0.0376 (11)	0.0423 (12)	0.0138 (10)	0.0216 (10)	0.0224 (10)
O3	0.0404 (8)	0.0427 (8)	0.0511 (9)	0.0215 (7)	0.0308 (7)	0.0324 (8)
C29	0.0345 (10)	0.0341 (10)	0.0416 (11)	0.0129 (8)	0.0216 (9)	0.0172 (9)

### Geometric parameters (Å, °)

**Table:**

O1—C1	1.355 (2)	C16—C21	1.394 (2)
O1—H1O1	0.97 (3)	C16—C17	1.399 (2)
N1—C7	1.334 (2)	C17—C18	1.389 (3)
N1—C8	1.377 (2)	C17—H17A	0.9300
N2—C13	1.326 (2)	C18—C19	1.386 (3)
N2—C14	1.358 (2)	C18—H18A	0.9300
N3—C12	1.324 (2)	C19—C20	1.392 (3)
N3—C11	1.361 (2)	C19—H19A	0.9300
N4—C7	1.370 (2)	C20—C21	1.390 (3)
N4—C15	1.374 (2)	C20—H20A	0.9300
N4—H1N4	0.97 (3)	C21—H21A	0.9300
C1—C2	1.395 (2)	C22—C27	1.392 (2)
C1—C6	1.412 (2)	C22—C23	1.393 (2)
C2—C3	1.379 (3)	C23—C24	1.385 (2)
C2—H2A	0.9300	C23—H23A	0.9300
C3—C4	1.394 (2)	C24—C25	1.380 (3)
C3—H3A	0.9300	C24—H24A	0.9300
C4—C5	1.385 (2)	C25—C26	1.392 (3)
C4—H4A	0.9300	C25—H25A	0.9300
C5—C6	1.398 (2)	C26—C27	1.391 (2)
C5—H5A	0.9300	C26—H26A	0.9300
C6—C7	1.455 (2)	C27—H27A	0.9300
C8—C15	1.399 (2)	O2—C28	1.416 (3)
C8—C9	1.414 (2)	O2—H1O2	1.0039
C9—C10	1.364 (2)	C28—H28A	0.9600
C9—H9A	0.9300	C28—H28B	0.9600
C10—C11	1.428 (2)	C28—H28C	0.9600
C10—H10A	0.9300	O3—C29	1.407 (2)
C11—C14	1.421 (2)	O3—H1O3	0.9522
C12—C13	1.438 (2)	C29—H29A	0.9600
C12—C16	1.486 (2)	C29—H29B	0.9600
C13—C22	1.486 (2)	C29—H29C	0.9600
C14—C15	1.410 (2)		
			
Cg1···Cg1^i^	3.7885 (10)	Cg3···Cg4^i^	3.6348 (11)
Cg2···Cg3^i^	3.5234 (7)		
			
C1—O1—H1O1	104.8 (17)	C8—C15—C14	121.14 (15)
C7—N1—C8	105.76 (13)	C21—C16—C17	119.42 (16)
C13—N2—C14	117.55 (14)	C21—C16—C12	121.54 (15)
C12—N3—C11	118.73 (14)	C17—C16—C12	119.04 (15)
C7—N4—C15	106.63 (14)	C18—C17—C16	120.17 (17)
C7—N4—H1N4	127.7 (14)	C18—C17—H17A	119.9
C15—N4—H1N4	125.4 (14)	C16—C17—H17A	119.9
O1—C1—C2	117.77 (15)	C19—C18—C17	120.15 (19)
O1—C1—C6	122.16 (15)	C19—C18—H18A	119.9
C2—C1—C6	120.07 (15)	C17—C18—H18A	119.9
C3—C2—C1	119.88 (16)	C18—C19—C20	120.00 (18)
C3—C2—H2A	120.1	C18—C19—H19A	120.0
C1—C2—H2A	120.1	C20—C19—H19A	120.0
C2—C3—C4	120.86 (16)	C21—C20—C19	120.10 (19)
C2—C3—H3A	119.6	C21—C20—H20A	119.9
C4—C3—H3A	119.6	C19—C20—H20A	119.9
C5—C4—C3	119.55 (16)	C20—C21—C16	120.13 (18)
C5—C4—H4A	120.2	C20—C21—H21A	119.9
C3—C4—H4A	120.2	C16—C21—H21A	119.9
C4—C5—C6	120.87 (16)	C27—C22—C23	119.41 (15)
C4—C5—H5A	119.6	C27—C22—C13	119.70 (14)
C6—C5—H5A	119.6	C23—C22—C13	120.88 (15)
C5—C6—C1	118.74 (15)	C24—C23—C22	120.17 (16)
C5—C6—C7	122.07 (15)	C24—C23—H23A	119.9
C1—C6—C7	119.18 (15)	C22—C23—H23A	119.9
N1—C7—N4	111.95 (14)	C25—C24—C23	120.52 (16)
N1—C7—C6	122.72 (14)	C25—C24—H24A	119.7
N4—C7—C6	125.33 (15)	C23—C24—H24A	119.7
N1—C8—C15	109.29 (14)	C24—C25—C26	119.76 (16)
N1—C8—C9	129.16 (15)	C24—C25—H25A	120.1
C15—C8—C9	121.56 (15)	C26—C25—H25A	120.1
C10—C9—C8	118.49 (15)	C27—C26—C25	119.98 (17)
C10—C9—H9A	120.8	C27—C26—H26A	120.0
C8—C9—H9A	120.8	C25—C26—H26A	120.0
C9—C10—C11	120.76 (15)	C26—C27—C22	120.16 (16)
C9—C10—H10A	119.6	C26—C27—H27A	119.9
C11—C10—H10A	119.6	C22—C27—H27A	119.9
N3—C11—C14	119.68 (14)	C28—O2—H1O2	101.2
N3—C11—C10	118.80 (15)	O2—C28—H28A	109.5
C14—C11—C10	121.51 (15)	O2—C28—H28B	109.5
N3—C12—C13	120.96 (14)	H28A—C28—H28B	109.5
N3—C12—C16	116.40 (14)	O2—C28—H28C	109.5
C13—C12—C16	122.65 (14)	H28A—C28—H28C	109.5
N2—C13—C12	121.33 (14)	H28B—C28—H28C	109.5
N2—C13—C22	116.56 (14)	C29—O3—H1O3	108.4
C12—C13—C22	122.09 (14)	O3—C29—H29A	109.5
N2—C14—C15	121.75 (15)	O3—C29—H29B	109.5
N2—C14—C11	121.71 (15)	H29A—C29—H29B	109.5
C15—C14—C11	116.51 (14)	O3—C29—H29C	109.5
N4—C15—C8	106.38 (14)	H29A—C29—H29C	109.5
N4—C15—C14	132.46 (15)	H29B—C29—H29C	109.5
			
O1—C1—C2—C3	179.52 (16)	N3—C11—C14—N2	−2.1 (2)
C6—C1—C2—C3	−0.7 (3)	C10—C11—C14—N2	178.68 (15)
C1—C2—C3—C4	−0.5 (3)	N3—C11—C14—C15	179.95 (14)
C2—C3—C4—C5	1.1 (3)	C10—C11—C14—C15	0.7 (2)
C3—C4—C5—C6	−0.5 (3)	C7—N4—C15—C8	0.49 (17)
C4—C5—C6—C1	−0.6 (3)	C7—N4—C15—C14	−177.94 (17)
C4—C5—C6—C7	178.09 (16)	N1—C8—C15—N4	−0.57 (18)
O1—C1—C6—C5	−179.00 (15)	C9—C8—C15—N4	179.39 (15)
C2—C1—C6—C5	1.3 (2)	N1—C8—C15—C14	178.09 (15)
O1—C1—C6—C7	2.3 (2)	C9—C8—C15—C14	−2.0 (3)
C2—C1—C6—C7	−177.50 (15)	N2—C14—C15—N4	1.1 (3)
C8—N1—C7—N4	−0.09 (19)	C11—C14—C15—N4	179.09 (17)
C8—N1—C7—C6	179.25 (15)	N2—C14—C15—C8	−177.11 (15)
C15—N4—C7—N1	−0.26 (19)	C11—C14—C15—C8	0.8 (2)
C15—N4—C7—C6	−179.58 (15)	N3—C12—C16—C21	129.81 (17)
C5—C6—C7—N1	177.68 (16)	C13—C12—C16—C21	−50.0 (2)
C1—C6—C7—N1	−3.6 (2)	N3—C12—C16—C17	−49.6 (2)
C5—C6—C7—N4	−3.1 (3)	C13—C12—C16—C17	130.63 (17)
C1—C6—C7—N4	175.63 (15)	C21—C16—C17—C18	1.4 (3)
C7—N1—C8—C15	0.41 (18)	C12—C16—C17—C18	−179.21 (17)
C7—N1—C8—C9	−179.54 (17)	C16—C17—C18—C19	−0.9 (3)
N1—C8—C9—C10	−178.63 (17)	C17—C18—C19—C20	−0.4 (3)
C15—C8—C9—C10	1.4 (3)	C18—C19—C20—C21	1.3 (3)
C8—C9—C10—C11	0.2 (3)	C19—C20—C21—C16	−0.8 (3)
C12—N3—C11—C14	1.3 (2)	C17—C16—C21—C20	−0.6 (3)
C12—N3—C11—C10	−179.45 (15)	C12—C16—C21—C20	−179.91 (17)
C9—C10—C11—N3	179.52 (16)	N2—C13—C22—C27	−50.5 (2)
C9—C10—C11—C14	−1.3 (3)	C12—C13—C22—C27	131.08 (17)
C11—N3—C12—C13	0.7 (2)	N2—C13—C22—C23	128.11 (17)
C11—N3—C12—C16	−179.14 (14)	C12—C13—C22—C23	−50.3 (2)
C14—N2—C13—C12	1.3 (2)	C27—C22—C23—C24	0.7 (3)
C14—N2—C13—C22	−177.07 (14)	C13—C22—C23—C24	−177.91 (16)
N3—C12—C13—N2	−2.1 (2)	C22—C23—C24—C25	−1.1 (3)
C16—C12—C13—N2	177.70 (15)	C23—C24—C25—C26	0.4 (3)
N3—C12—C13—C22	176.20 (15)	C24—C25—C26—C27	0.7 (3)
C16—C12—C13—C22	−4.0 (2)	C25—C26—C27—C22	−1.0 (3)
C13—N2—C14—C15	178.55 (15)	C23—C22—C27—C26	0.3 (3)
C13—N2—C14—C11	0.7 (2)	C13—C22—C27—C26	178.97 (16)

Symmetry codes: (i) −*x*+1, −*y*, −*z*+1.

### Hydrogen-bond geometry (Å, °)

**Table:**

*D*—H···*A*	*D*—H	H···*A*	*D*···*A*	*D*—H···*A*
O2—H1O2···O3	1.00	1.71	2.700 (3)	167
O3—H1O3···N3^ii^	0.95	1.87	2.814 (2)	172
N4—H1N4···O2	0.97 (3)	1.78 (3)	2.750 (2)	177 (2)
O1—H1O1···N1	0.97 (4)	1.66 (4)	2.570 (2)	154 (3)
C2—H2A···O1^iii^	0.93	2.48	3.356 (3)	156
C5—H5A···O2	0.93	2.42	3.310 (3)	160
C28—H28C···Cg5^iv^	0.96	2.95	3.534 (2)	120

Symmetry codes: (ii) *x*+1, *y*, *z*; (iii) −*x*+1, −*y*−1, −*z*+1; (iv) −*x*+1, −*y*, −*z*.
